# Correlation between melphalan chemotherapy with longitudinal global strain indices of the left ventricle in multiple myeloma patients: A velocity vector imaging (VVI) echocardiography study

**DOI:** 10.22088/cjim.15.1.25

**Published:** 2024

**Authors:** Liaosadat Mirsafaee, Mohammad Dehghani Firouzabadi, Sayeh Parkhideh, Hassan Vahidnezhad, Fatemeh Dehghani Firouzabadi, Maryam Arab, David M. Yousem, Mersedeh Karvandi

**Affiliations:** 1 Department of Cardiology, Ramsar Campus, Mazandaran University of Medical Sciences, Sari, Iran; 2 Endocrinology and Metabolism Research Center (EMRC), Vali-Asr Hospital, School of Medicine, Tehran University of Medical Sciences, Tehran, Iran; 3 ENT and Head & Neck Research Center, the Five Senses Institute, Iran University of Medical Sciences, Tehran, Iran; 4 Hematopoietic Stem Cell Research Center (HSCRC), Shahid Beheshti University of Medical Sciences, Tehran, Iran; 5 Jefferson Institute of Molecular Medicine, Sidney Kimmel Medical College, Thomas Jefferson University, Philadelphia, Pennsylvania, USA; 6 Department of Radiology, Boston Children’s Hospital, Harvard Medical School, Boston, MA, USA; 7 Russel H. Morgan Department of Radiology and Radiological Science, John Hopkin’s, Medical Institution, Baltimore, MD, USA; 8 Cardivascular Research Institute of Taleghani Hospital, Shahid Beheshti University of Medical Sciences, Tehran, Iran

**Keywords:** Melphalan chemotherapy, multiple myeloma, velocity vector imaging

**Letter to the editor,**

Multiple myeloma (MM) is characterized by the neoplastic proliferation of plasma cells, leading to the production of monoclonal immunoglobulins. High-dose chemotherapy with autologous stem cell transplant has been used in the treatment of patients with MM (1). Melphalan (L-phenylalanine nitrogen mustard) is one of the most effective chemotherapeutic drugs in the treatment of MM (2). The side effects of melphalan include anemia, nausea, vomiting, allergic reactions, oral ulcers, nephrotoxicity, and arrhythmia at high doses (3). The effect of this drug on cardiac mechanics and heart damage is not well established, as previous studies have only focused on the electrocardiographic and metabolic effects of this drug.

Velocity vector imaging (VVI) is a relatively new echocardiographic technique based on two-dimensional grayscale imaging (angle-dependent), which can provide more information about cardiac function than tissue Doppler imaging (4). Strain echocardiography is also a new non-invasive technique for the evaluation of global and segmental cardiac function. It measures the percentage change in myocardial length from a relaxed to a contractile state and assesses different spatial parts of contractile function in three directions, both globally and regionally. It is also used for the quantitative evaluation of intracellular desynchrony and different components of myocardial function, including longitudinal myocardial shortening, which is not otherwise easy to measure (5, 6). To evaluate the role of melphalan in myocardial function among patients with MM, we performed VVI on 18 MM patients undergoing melphalan therapy. We used VVI to measure 16 segments of the left ventricle (LV) in three consecutive cardiac cycles to determine the global longitudinal strain. 

**Figure 1:**
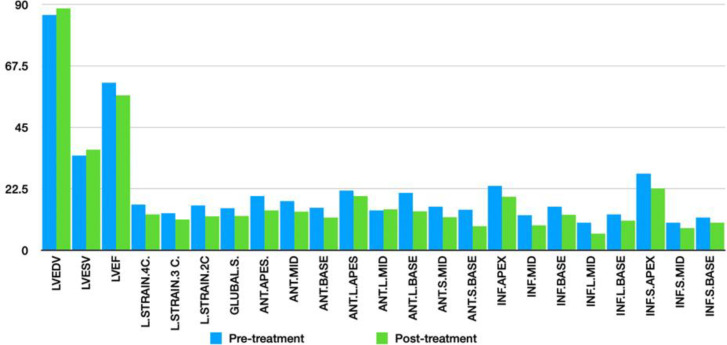
Strain echocardiographic findings before and after treatment with melphalan

The results showed that the left ventricular ejection fraction and global longitudinal strain significantly decreased after melphalan therapy (p<0.001). There was also a remarkable decrease in the contractile function of all LV segments, except for the anterolateral myocardial wall (p<0.001 for all) (table 1, figure 1). The left ventricular function was affected in different heart segments, but there was no significant difference in the left ventricular end-diastolic volume or left ventricular end-systolic volume before and after three weeks of melphalan therapy (P=0.318 and P=0.15, respectively).

Overall, for the first time, in this prospective observational study, we found a significant reduction in the LV function and LV ejection fraction after three weeks of single-agent melphalan treatment using VVI echocardiography. Further studies should be conducted on a larger set of patients to detect melphalan-induced heart damage and cardiotoxic effects. Corroboration of our findings by using other parameters, such as troponin, brain natriuretic peptide, and endothelin to increase the sensitivity in detecting heart damage would help advice patients when contemplating melphalan administration. 

**Table 1:** Strain echocardiographic findings before and after treatment with melphalan

**Parameters**	**Measurements**		**P-value**
	**Pre treatment**	**Post treatment**	
**LVEDV**	86.1±12.3	88.6±15.1	0.318
**LVESV**	34.6±11.6	36.8±7.4	0.150
**LVEF**	61.3±5.4	56.7±3.9	<0.001
**L.strain.4C.**	16.7±3.1	13.1±2.5	0.012
**L.strain.3 C.**	13.5±4.4	11.2±2.1	<0.001
**L.strain.2C**	16.4±2.3	12.4±2.3	<0.001
**Glubal.S.**	15.4±2.5	12.5±1.5	<0.001
**Ant.apes.**		19.8±5.7	14.5±3.3	<0.001
**Ant.mid**	18±4.4	14.1±3.9	<0.001
**Ant.base**	15.5±2.5	12±2.5	<0.001
**Ant.L.apes**	21.8±6.5	19.8±4.3	0.239
**Ant.L.mid**	14.5±5.3	15±3.2	0.623
**Ant.L.base**	21±9	14.3±4.2	<0.001
**Ant.S.mid**	16±3.8	12.1±5	0.002
**Ant.S.base**	14.8±5.6	8.8±3.2	<0.001
**Inf.apex**	23.6±6.1	19.6±4	<0.001
**Inf.mid**	12.8±4.5	9±4.6	<0.001
**Inf.base**	16±5.6	13±5.4	<0.001
**Inf.L.mid**	10±2.4	6.1±1	<0.001
**Inf.L.base**	13.1±6.6	10.8±4.7	<0.001
**Inf.S.apex**	28±3.6	22.5±6.9	<0.001
**Inf.S.mid**	10±5.4	8±5	<0.001
**Inf.S.base**	12±5.3	10±5.9	<0.001

LVEDV: Left ventricular end-diastolic volume; LVESV: Left ventricular end-systolic volume; LVEF: Left ventricular ejection fraction; L.strain.4C.: Longitudinal strain in the 4-chamber view; L.strain.3C.: Longitudinal strain in the 3-chamber view; L.strain.2C: Longitudinal strain in the 2-chamber view; Global.S.: Global longitudinal strain; Ant.apes.: Anterior apical segment; Ant.mid: Anterior mid-segment; Ant.base: Anterior basal segment; Ant.L.apes: Anterolateral apical segment; Ant.L.mid: Anterolateral mid-segment; Ant.L.base: Anterolateral basal segment; Ant.S.mid: Anteroseptal mid-segment; Ant.S.base: Anteroseptal basal segment; Inf.apex: Inferior apical segment; Inf.mid: Inferior mid-segment; Inf.base: Inferior basal segment; Inf.L.mid: Inferolateral mid-segment; Inf.L.base: Inferolateral basal segment; Inf.S.apex: Inferoseptal apical segment; Inf.S.mid: Inferoseptal mid-segment; Inf.S.base: Inferoseptal basal segment.
